# Investigation of the Clinical Significance and Prognostic Value of the lncRNA *ACVR2B-As1* in Liver Cancer

**DOI:** 10.1155/2019/4602371

**Published:** 2019-10-30

**Authors:** Yuanyuan Nie, Yan Jiao, Yanqing Li, Wei Li

**Affiliations:** ^1^Stem Cell and Cancer Center, First Hospital, Jilin University, Changchun, Jilin 130061, China; ^2^Department of Hepatobiliary and Pancreatic Surgery, The First Hospital of Jilin University, Changchun, Jilin 130021, China; ^3^Department of Pathophysiology, College of Basic Medical Sciences, Jilin University, Changchun, Jilin 130021, China

## Abstract

A refined liver cancer staging system and effective prognostic prediction can help clinicians make optimized treatment decisions, which is essential in our fight against cancer and for improving the unsatisfying survival rate of liver cancer globally. The prognosis of liver cancer is not only related to tumor status, it is also affected by the patients' liver functions and the chosen treatment. Currently, several staging systems are being tested. Herein, we analyzed RNA-seq data from the TCGA database and identified a newly annotated lncRNA, *ACVR2B-AS1*, whose expression is upregulated in liver cancer. Higher *ACVR2B-AS1* expression is an independent adverse prognostic factor for overall survival (OS) and relapse-free survival (RFS) in liver cancer patients. Our work suggests that the lncRNA *ACVR2B-AS1* could be a candidate biomarker for liver cancer prognosis. Furthermore, *ACVR2B-AS1* might serve as a potential therapeutic target, which is a possibility that is worthy of further study.

## 1. Introduction

Liver cancer is one of the leading causes of cancer-related deaths worldwide [1]. Although numerous resources have been invested into basic research and clinical trials, the survival rate of liver cancer patients remains dismal. For example, in the US, the latest five-year relative survival rate for liver cancer patients is only 18% [2]. This low rate is largely due to the aggressive nature of liver cancer and the fact that most patients diagnosed are already at advanced stages [3]. While the current staging system utilizes both patient assessment and proposed treatment responses, it remains important to refine the risk classification system, which is crucial for optimizing decision making and reducing cancer mortality [1].

Long noncoding RNAs (lncRNAs) represent a class of genes that are not translated into proteins and whose functions have only begun to emerge in the past few years [4, 5]. They are evolutionarily less conserved than coding genes, and it is estimated that the number of lncRNA genes is far larger than that of protein coding genes [5], although their exact abundance is not clear. Increasing evidence has demonstrated that lncRNAs have important biological functions and that their dysregulation is associated with diseases, including cancers [6]. However, the majority of annotated lncRNAs have not been functionally characterized, and their biological significance remains elusive [6].

*ACVR2B-AS1* (*ACVR2B-antisense RNA1*) is a recently annotated lncRNA [7]. It is located on 3p22.2 and is transcribed from the opposite strand of *ACVR2B*. The function of *ACVR2B-AS1* has not been studied. Here, using data extracted from the TCGA database, we identified *ACVR2B-AS1* as an independent prognostic factor for liver cancer patient clinical outcomes. This may help us to refine liver cancer prognostic prediction and to further studies of *ACVR2B-AS1*.

## 2. Materials and Methods

### 2.1. Data Mining

RNA-seq data and patients' clinical characteristics were downloaded from the TCGA database (https://portal.gdc.cancer.gov/). Transcript abundances were quantified using the RNA-Seq data via the Expectation-Maximization (RSEM) software [8].

### 2.2. Statistical Analysis

All statistical tests were performed in R [9]. Nonparametric Wilcoxon and Kruskal–Wallis tests were used for differential expression analysis among different subgroups. The diagnostic value of *ACVR2B-AS1* was estimated using a receiver operating characteristic (ROC) curve drawn with the pROC package [10], and the area under curve (AUC) was calculated as previously described [10]. A cut-off value was determined by utilizing the ROC curve, and the overall population was then divided into two groups (*ACVR2B-AS1* High and *ACVR2B-AS1* Low) for the subsequent analysis. The associations between *ACVR2B-AS1* and patient clinical features were analyzed via Fisher's exact or Chi-squared tests. Overall survival and relapse-free survival were determined via Kaplan–Meier curves using the survival package in R [11]. The statistical significance of the differences noticed above was calculated using the log-rank test. The prognostic capabilities of *ACVR2B-AS1* were assessed via univariate and multivariate Cox regression analysis using the Cox proportional hazard model. The ggplot2 package in R was used for data visualization [12].

## 3. Results

### 3.1. Patient Characteristics

RNA-seq data from a total of 371 patients diagnosed with liver cancer were extracted from the TCGA database for analysis. The clinical characteristics of the patients, including age, sex, tumor histological type, grade, stage, and vital status, are listed in Table 1.

### 3.2. ACVR2B-AS1 Expression Is Upregulated in Liver Cancer

**Figure 1:**
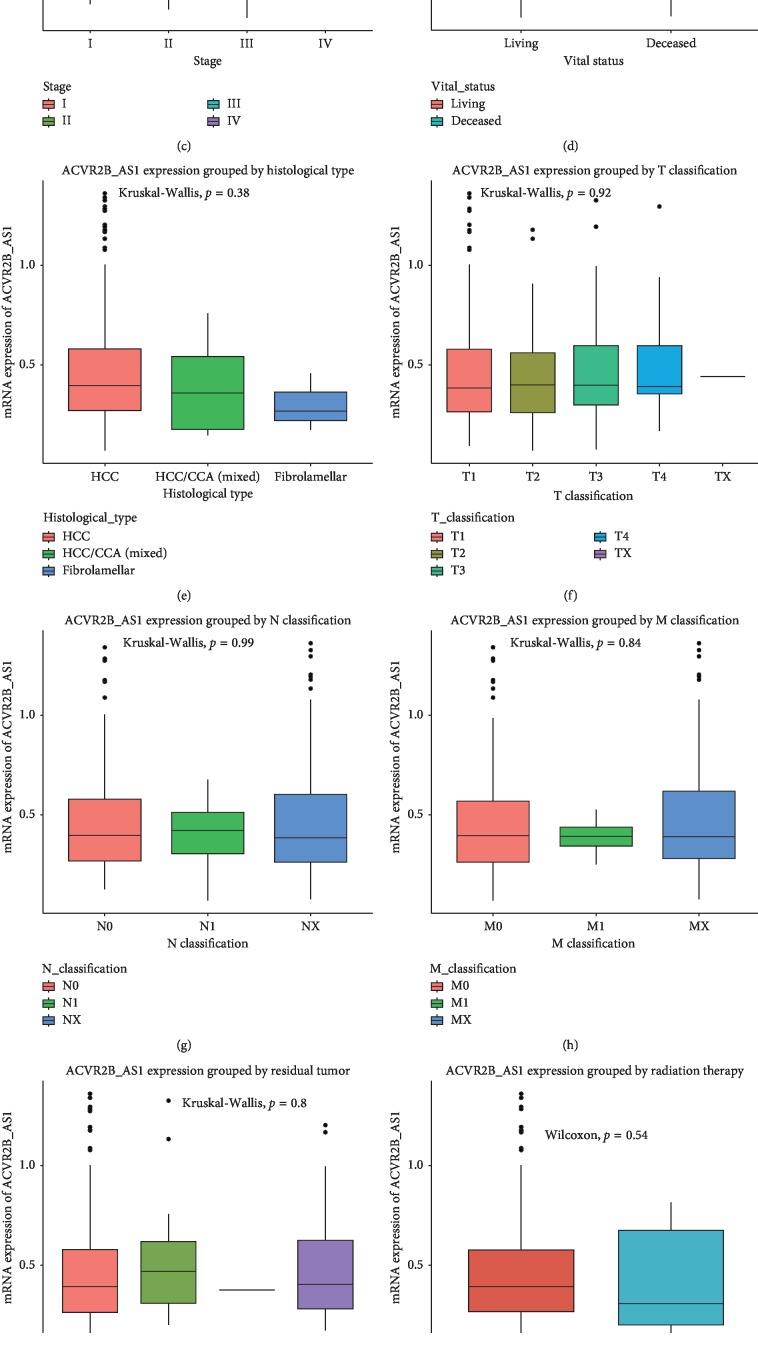
*ACVR2B-AS1* is overexpressed in liver cancer and is differentially expressed in the corresponding subtypes. The significance was calculated based on nonparametric Wilcoxon and Kruskal–Wallis tests. Note: the subgroups include tumors versus normal liver tissue, histological grade, stage, vital status, histological type, T classification, N classification, M classification, residual tumor, radiation therapy, age, and sex.

*ACVR2B-AS1* levels were significantly elevated in liver cancer specimens compared with their normal counterparts (*p*=0.034) (Figure 1). A subgroup analysis revealed that *ACVR2B-AS1* was differentially expressed in tumors of different histological grades (*p*=0.02), and grade IV tumors showed the highest *ACVR2B-AS1* levels. Furthermore, by categorizing the patients based on vital status, we found that deceased patients showed higher *ACVR2B-AS1* expression levels than did patients who were still alive at the time of sampling (*p*=0.0043). No significant differential expression was detected in the other subgroups analyzed.

**Figure 2:**
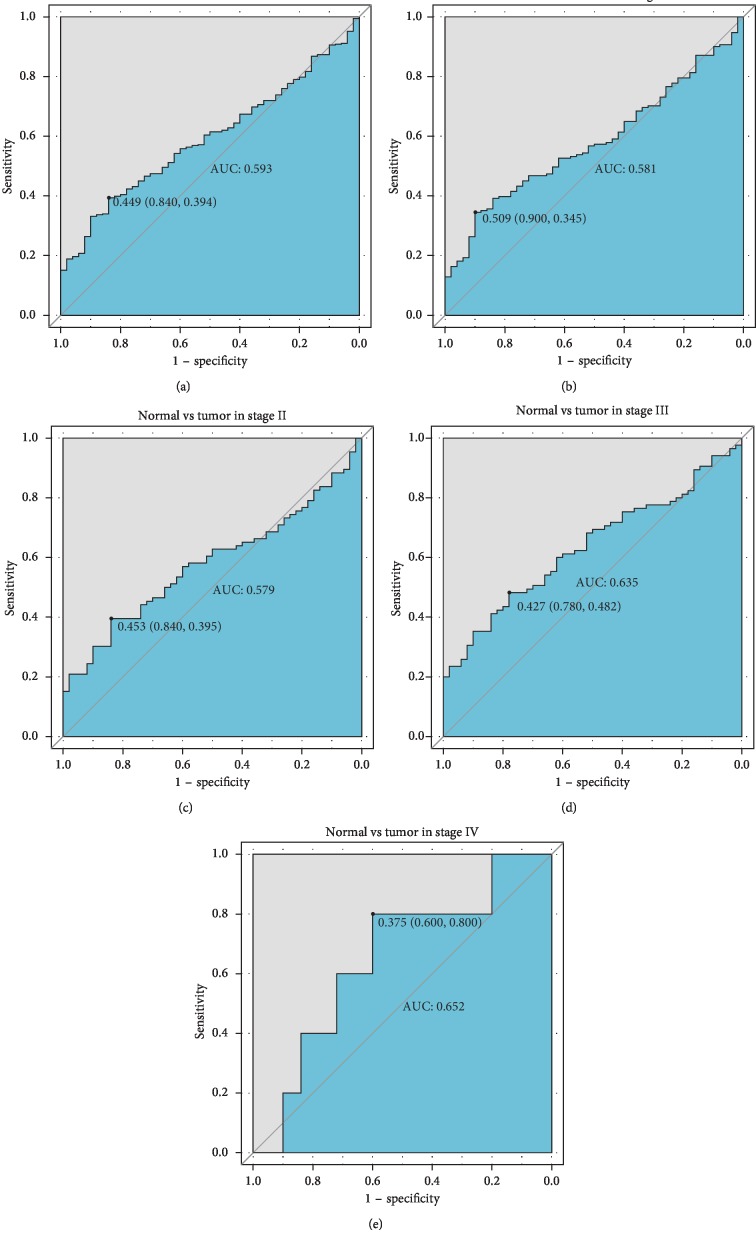
The ROC curves of *ACVR2B-AS1* in liver cancer cohorts and different stages. Abbreviations: AUC, area under the curve.

To further examine these findings, we generated an ROC curve to test the diagnostic abilities of *ACVR2B-AS1* level for liver cancer diagnosis and histological grade classification. The results of the AUC analysis did not show any significance (Figure 2), indicating that *ACVR2B-AS1* alone may not be an accurate diagnostic parameter.

### 3.3. ACVR2B-AS1 Levels Correlate with Patient Vital Status

Consistent with the previous results, correlation analysis confirmed that the vital status of the patients is correlated with *ACVR2B-AS1* levels (*p*=0.003) (Table 2). The correlation between *ACVR2B-AS1* levels and histological grade hardly reached statistical significance (*p*=0.054).

### 3.4. ACVR2B-AS1 Is an Independent Adverse Prognostic Factor for OS in Liver Cancer

**Figure 3:**
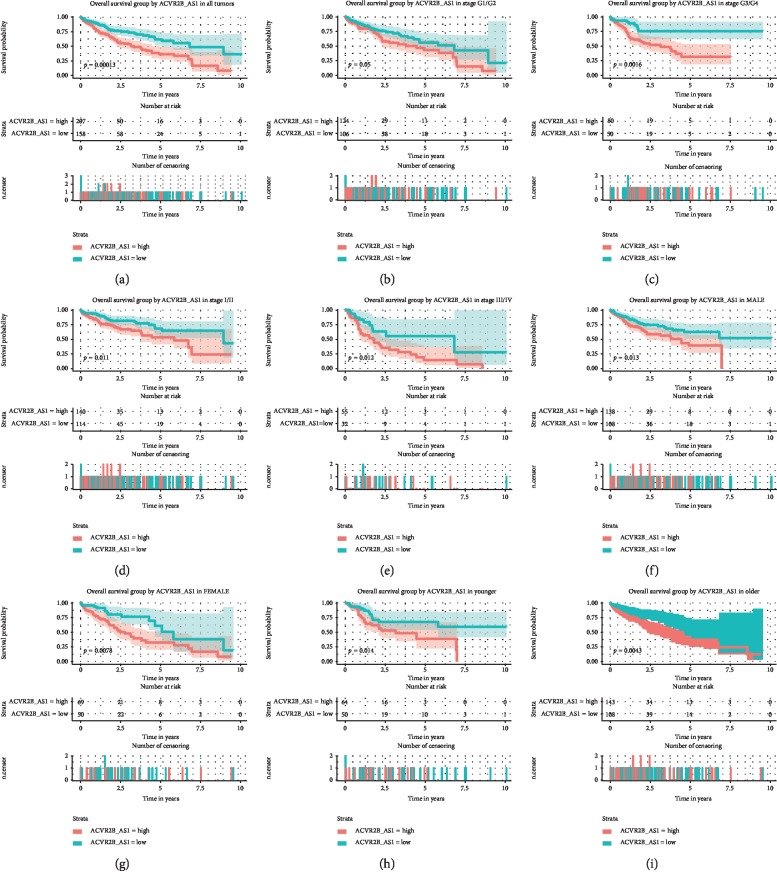
Overall survival outcomes based on *ACVR2B-AS1* expression levels in different subgroups. Notes: The subgroups include tumor grades G1/G2, G3/G4, stage I/II, stage III/IV, males, females, and younger and older patients.

We next sought to verify the prognostic usefulness of *ACVR2B-AS1* in liver cancer. A Kaplan–Meier curve was plotted against different *ACVR2B-AS1* levels within the different subgroups (previously defined via the ROC curve against vital status) (Figure 3). The log-rank test revealed that patients with lower *ACVR2B-AS1* expression levels had a significantly longer overall survival (OS) than those with higher *ACVR2B-AS1* expression levels (*p*=0.0013). A subgroup analysis showed that *ACVR2B-AS1*-low patients displayed better OS among all of the subgroups analyzed.

To better verify these results, we also performed a Cox regression analysis (Table 3). A univariate Cox regression analysis showed that patients with high *ACVR2B-AS1* expression levels had significantly poorer outcomes (HR = 2.03, 95% CI (1.4–2.94)) compared with those with low *ACVR2B-AS1* expression levels. Other common clinical parameters were also analyzed, and tumor clinical stage, together with T classification, and residual tumor were shown to be unfavorable factors for patient survival. To rule out possible interference among the variables, we performed a multivariate Cox regression analysis, which confirmed that the adverse effects of high *ACVR2B-AS1* expression, T classification, and residual tumor remained significant. Together, these data demonstrated that high *ACVR2B-AS1* expression (HR = 1.90, 95% CI (1.31–2.76)) is an independent prognostic factor for OS in liver cancer patients.

### 3.5. High ACVR2B-AS1 Expression Predicts Shorter RFS in Liver Cancer

**Figure 4:**
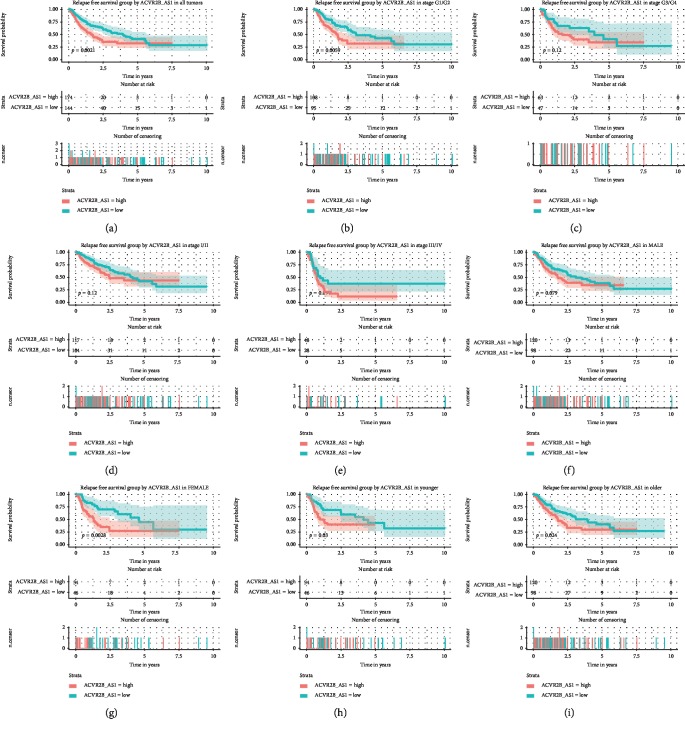
Relapse-free survival outcomes based on different *ACVR2B-AS1* expression levels in different subgroups. Notes: The subgroups include tumor grades G1/G2, G3/G4, stage I/II, stage III/IV, males, females, and younger and older patients.

We next evaluated the prognostic value of *ACVR2B-AS1* expression level for relapse-free survival (RFS) (Figure 4). The log-rank test showed that patients with high *ACVR2B-AS1* expression levels had significantly shorter RFS (*p*=0.0021). When we analyzed this parameter within the different categories, we found that although high *ACVR2B-AS1* expression levels predicted poorer RFS in patients at histological stage I (*p*=0.0059), this effect failed to reach statistical significance in stage III patients (*p*=0.12). There were no significant differences in RFS between high and low *ACVR2B-AS1* patients in either the stage I or stage III groups, respectively. It was interesting to see that when taking sex into consideration, female patients with higher *ACVR2B-AS1* expression levels had much shorter RFS than those females with lower expression levels (*p*=0.0028), although no difference was found in males. In both the young and old subgroups, patients with higher *ACVR2B-AS1* expression levels had shorter times before relapse.

Consistent with the aforementioned findings, a univariate Cox regression analysis showed that high *ACVR2B-AS1* expression is an unfavorable prognostic factor for RFS (HR = 1.71, 95% CI [1.21–2.41]) (Table 4). An additional multivariate Cox analysis confirmed that *ACVR2B-AS1* is an independent adverse prognostic factor for RFS, and the adjusted hazard ratio was 1.66 (*p*=0.005).

## 4. Discussion

Liver cancer is one of the most life threatening cancers, and most people are diagnosed at later stages when surgical resection is not an option [3]. A refined staging system and more effective prognostic assessment is not only necessary for the selection of the best treatment for individual patients, but also important for designing clinical trials and coordinating the exchange of information between researchers with comparable criteria [1]. The old TNM staging system has gone out of fashion because establishing prognosis in liver cancer patients is highly sensitive to the patients' liver functions, the chosen treatment, and other factors. Although there are several staging systems currently being tested, most of which include tumor status, liver function, patient status, and treatment responses, a more comprehensive system is urgently needed. Our previous work revealed several RNA biomarkers that are associated with cancer prognosis and can help us refine cancer staging systems [13–18]. Here, using data extracted from the TCGA database, we identified a novel lncRNA, *ACVR2B-AS1*, whose upregulation is common in liver cancer, and found that higher *ACVR2B-AS1* expression predicted poorer OS and RFS in liver cancer patients.

*ACVR2B-AS1* is a newly annotated lncRNA. So far, only two studies using TCGA data have mentioned potential roles of *ACVR2B-AS1* in cancers. One of these studies that focused on endometrial cancer (UCEC) found that *ACVR2B-AS1* was upregulated in cancer samples. A combined lncRNA-focus expression signature (LFES) that included 10 additional lncRNAs functioned as a superior unfavorable prognostic factor (AUC = 0.887) [19]. The other study, however, identified *ACVR2B-AS1* as an independent favorable prognostic factor in breast cancer [20]. Our study revealed that *ACVR2B-AS1* is an adverse factor in liver cancer. It is possible that *ACVR2B-AS1* may have different roles in distinct biological contexts.

Although controversial results have been reported from genome-wide studies indicating possible regulatory roles of *ACVR2B-AS1*, its functions have not been experimentally dissected [19, 20]. Recent accumulating evidence has indicated that neighboring/overlapping genes tend to show correlated expression in eukaryotic genomes, indicating possible local regulatory mechanisms [21, 22]. Thus, as *ACVR2B-AS1* is an intragenic antisense long noncoding RNA that overlaps with the promoter region of the *ACVR2B* protein coding gene, it is possible that *ACVR2B-AS1* might regulate its neighbor gene *ACVR2B* in a cis manner. For example, *ACVR2B-AS1* transcription might disturb the activation of *ACVR2B* transcription via “transcription interference” [23]. Alternatively, *ACVR2B-AS1* activation might promote the transition of the local chromatin into a relatively open state to enhance *ACVR2B* transcription [24]. *ACVR2B* encodes a protein called activin receptor type IIB, which is a major receptor of the transforming growth factor beta (TGF-*β*) signaling pathway with many functions. For example, binding of *ACVR2B* to its ligand activin can activate the transcription of genes that inhibit muscle growth and is probably responsible for cancer-related cachexia [25, 26]. *ACVR2B* has also been reported to be a member of the MALAT1/miR-194-5p/*ACVR2B* signaling axis, which promotes clear cell kidney carcinoma (KIRC) progression [27]. In our study, *ACVR2B-AS1* was associated with poorer prognosis, although it remains unknown if and how *ACVR2B-AS1* might regulate tumorigenesis and progression via the mechanisms we mentioned above. Further experiments are required to answer these remaining questions.

It was worth noting that liver functions, another prognostic factor, are not included into the multivariate COX regression analysis, since such data are not available in the TCGA database. Nonetheless, our work here has demonstrated the prognostic value of ACVR2B-AS1 in multivariate COX regression analysis and managed to raise the potential possibility of incorporating *ACVR2B-AS1* into liver cancer staging. To better testify its independent value as a prognosticator, further studies are encouraged, for example, in another patients cohort where more detailed information is available.

## 5. Conclusions

Overall, our data reported here identified the lncRNA *ACVR2B-AS1* as an independent adverse prognostic factor in liver cancer. Attempts to decipher and identify the clinical significance of the enormous information hidden in RNA-seq data and to characterize the roles of *ACVR2B-AS1* are encouraged, which might help refine the staging system in the future, especially at a time when RNA-seq are becoming more prevalent and more data will become available. In addition, more attention should be paid to basic research on *ACVR2B-AS1*, which might be a potential therapeutic target for liver cancer.

## Figures and Tables

**Table 1:** Clinical characteristic of the included patients.

Characteristics	Number of pts (%)
*Age*	
<55	117 (31.62)
≥55	253 (68.38)
NA	1 (0)

*Gender*	
Female	121 (32.61)
Male	250 (67.39)

*Histological type*	
Fibrolamellar carcinoma	3 (0.81)
Hepatocellular carcinoma	361 (97.3)
Hepatocholangiocarcinoma	7 (1.89)

*Histologic grade*	
G1	55 (14.82)
G2	177 (47.71)
G3	122 (32.88)
G4	12 (3.23)
NA	5 (1.35)

*Stage*	
I	171 (46.09)
II	86 (23.18)
III	85 (22.91)
IV	5 (1.35)
NA	24 (6.47)

*T classification*	
T1	181 (48.79)
T2	94 (25.34)
T3	80 (21.56)
T4	13 (3.5)
TX	1 (0.27)
NA	2 (0.54)

*N classification*	
N0	252 (67.92)
N1	4 (1.08)
NX	114 (30.73)
NA	1 (0.27)

*M classification*	
M0	266 (71.7)
M1	4 (1.08)
MX	101 (27.22)

*Radiation therapy*	
No	338 (91.11)
Yes	8 (2.16)
NA	25 (6.74)

*Residual tumor*	
R0	324 (87.33)
R1	17 (4.58)
R2	1 (0.27)
RX	22 (5.93)
NA	7 (1.89)

*Vital status*	
Deceased	130 (35.04)
Living	241 (64.96)

*Relapse*	
No	179 (48.25)
Yes	139 (37.47)
NA	53 (14.29)

*ACVR2B-AS1*	
High	211 (56.87)
Low	160 (43.13)

**Table 2:** Correlation between the clinicopathologic variables and *ACVR2B-AS1* expression.

Clinical characteristics	*ACVR2B-AS1* expression							
	Variable	No. of patients	High	%	Low	%	*χ* ^2^	*p* value
Age	<55	117	67	(31.75)	50	(31.45)	0	1.000
	≥55	253	144	(68.25)	109	(68.55)		

Gender	Female	121	71	(33.65)	50	(31.25)	0.1417	0.6557
	Male	250	140	(66.35)	110	(68.75)		

Histological type	Fibrolamellar	3	1	(0.47)	2	(1.25)	1.2706	0.515
	Hepatocellular	361	207	(98.1)	154	(96.25)		
	Hepatocholangiocarcinoma	7	3	(1.42)	4	(2.5)		

Histologic grade	G1	55	28	(13.46)	27	(17.09)	7.2563	0.054
	G2	177	97	(46.63)	80	(50.63)		
	G3	122	72	(34.62)	50	(31.65)		
	G4	12	11	(5.29)	1	(0.63)		

Stage	I	171	92	(46.23)	79	(53.38)	2.8209	0.458
	II	86	50	(25.13)	36	(24.32)		
	III	85	53	(26.63)	32	(21.62)		
	IV	5	4	(2.01)	1	(0.68)		

T Classification	T1	181	97	(46.19)	84	(52.83)	2.7575	0.647
	T2	94	56	(26.67)	38	(23.9)		
	T3	80	47	(22.38)	33	(20.75)		
	T4	13	9	(4.29)	4	(2.52)		
	TX	1	1	(0.48)	0	(0)		

N classification	N0	252	147	(70)	105	(65.62)	1.588	0.528
	N1	4	3	(1.43)	1	(0.62)		
	NX	114	60	(28.57)	54	(33.75)		

M Classification	M0	266	154	(72.99)	112	(70)	1.1272	0.597
	M1	4	3	(1.42)	1	(0.62)		
	MX	101	54	(25.59)	47	(29.38)		

Radiation therapy	No	338	191	(98.45)	147	(96.71)	0.5046	0.307
	Yes	8	3	(1.55)	5	(3.29)		

Residual tumor	R0	324	183	(88.83)	141	(89.24)	0.8406	1.000
	R1	17	10	(4.85)	7	(4.43)		
	R2	1	1	(0.49)	0	(0)		
	RX	22	12	(5.83)	10	(6.33)		

Vital status	Deceased	130	88	(41.71)	42	(26.25)	8.8834	**0.003**
	Living	241	123	(58.29)	118	(73.75)		

**Table 3:** Univariate and multivariate analysis of overall survival in patients with liver cancer.

Parameters	Univariate analysis			Multivariate analysis		
	Hazard ratio	95%CI (lower∼upper)	*p* value	Hazard ratio	95% CI (lower-upper)	*p* value
Age	1.02	0.7–1.48	0.926			
Gender	0.82	0.57–1.16	0.263			
Histological type	0.98	0.27–3.63	0.982			
Histologic grade	1.05	0.85–1.31	0.651			
Stage	1.38	1.15–1.65	0.001	0.90	0.72–1.11	0.325
T Classification	1.65	1.38–1.98	≤0.001	1.72	1.36–2.16	≤0.001
N classification	0.71	0.5–1.03	0.071			
M Classification	0.70	0.48–1.02	0.061			
Radiation therapy	0.52	0.26–1.03	0.061			
Residual tumor	1.42	1.12–1.79	0.004	1.46	1.14–1.87	0.003
*ACVR2B-AS1*	2.03	1.4–2.94	≤0.001	1.90	1.31–2.76	0.001

**Table 4:** Univariate and multivariate analysis of relapse-free survival in patients with liver cancer.

Parameters	Univariate analysis			Multivariate analysis		
	Hazard ratio	95%CI (lower∼upper)	*p* value	Hazard ratio	95% CI (lower-upper)	*p* value
Age	0.89	0.63–1.27	0.521			
Gender	0.98	0.69–1.4	0.919			
Histological type	2.03	0.66–6.29	0.218			
Histologic grade	0.98	0.8–1.21	0.873			
Stage	1.66	1.38–1.99	≤0.001	1.16	0.9–1.5	0.259
T Classification	1.78	1.49–2.12	≤0.001	1.57	1.2–2.04	0.001
N classification	0.98	0.68–1.42	0.926			
M Classification	1.19	0.8–1.78	0.394			
Radiation therapy	0.75	0.26–2.17	0.592			
Residual tumor	1.27	1.01–1.61	0.042	1.39	1.09–1.76	0.007
*ACVR2B-AS1*	1.71	1.21–2.41	0.002	1.66	1.17–2.37	0.005

## Data Availability

The data used to support the findings of this study are included within the article.
